# Integrating an antimicrobial stewardship bundle into advanced urgent care sites

**DOI:** 10.1017/ash.2025.10178

**Published:** 2025-10-03

**Authors:** Bacil Kadi, Suhair Shawar, Sydney Agnello, Kelci Coe, Luca Delatore, John Ross Dutton, Elizabeth Rozycki

**Affiliations:** 1 Department of Pharmacy, The Ohio State University Wexner Medical Center, Columbus, OH, USA; 2 Department of Internal Medicine, Division of Infectious Diseases, The Ohio State University Wexner Medical Center, Columbus, OH, USA; 3 Department of Emergency Medicine, Medical Director of Urgent Care, The Ohio State University Wexner Medical Center, Columbus, OH, USA

## Abstract

**Objective:**

Urgent care locations remain a target for antimicrobial stewardship initiatives due to high rates of inappropriate antibiotic prescribing, particularly for respiratory tract infections. This study evaluated the impact of an antimicrobial stewardship bundle at urgent care sites on appropriate antibiotic prescriptions.

**Design:**

Retrospective, observational, pre- and post-analysis.

**Setting:**

Four advanced urgent care sites affiliated with an academic medical institution.

**Patients:**

Urgent care patients evaluated for respiratory tract infections.

**Intervention:**

An antimicrobial stewardship bundle including clinician-signed stewardship posters, over-the-counter medication prescription pads, and an educational campaign was introduced to advanced urgent care sites from October to November 2024. Respiratory tract infections were tiered by antibiotic appropriateness (1 = always appropriate; 2 = sometimes appropriate; 3 = never appropriate). Adults with tier 2 or 3 diagnoses from December 2023 to January 2024 (pre-group) and December 2024 to January 2025 (post-group) were included. The primary outcome was proportion of appropriate antibiotic prescriptions.

**Results:**

Two hundred and seventy-five patients with tier 2 and tier 3 indications were screened; 200 patients were included. Following implementation of the antimicrobial stewardship bundle, there was no significant difference in rates of appropriate antibiotic prescriptions for tier 2 and tier 3 respiratory infections between the pre- and post-groups (76% vs 74%, respectively; *P* = 0.68). After implementation, more patients received appropriate antibiotics for tier 2 indications (77% vs 95%; *p* = 0.13). The most prescribed antibiotic agents amongst both groups were amoxicillin-based antibiotics (15%) and azithromycin (15%).

**Conclusion:**

This study highlights challenges and opportunities for outpatient antimicrobial stewardship practices.

## Background

Antimicrobial resistance is a critical global health issue.^1^ The CDC launched its *Be Antibiotic Aware* campaign promoting core elements of antimicrobial stewardship with evidence-based initiatives improve antibiotic prescribing.^2^ Additionally, in 2020, the Joint Commission mandated outpatient antimicrobial stewardship in ambulatory care. Despite initiatives to increase antimicrobial stewardship, antibiotic-resistant infections have continued to grow by 20% between 2019 and 2022.^3^ In the United States, around 60% of antimicrobial cost is attributed to the outpatient setting, making it an important target for intervention.^4^ Among outpatient antibiotic prescriptions, it is estimated that around 30% are inappropriate based on diagnosis.^5,6^ With primarily viral etiologies, acute respiratory tract infections should be managed with supportive care; however, they continue to have high rates of inappropriate antibiotic prescribing, especially in urgent care settings.^7–9^

As part of the CDC initiatives targeting antimicrobial stewardship in the outpatient setting, there are four main categories: commitment, action for policy and practice, tracking and reporting, and education and expertise.^2,10,11^ Previous research and quality initiatives have implemented various stewardship interventions into outpatient practice sites to evaluate the effect on improving antibiotic prescribing in acute upper respiratory tract infections.^8,10,12–14^ However, an ideal strategy for outpatient antimicrobial stewardship has been difficult to determine. This study aimed to evaluate the impact of an antimicrobial stewardship bundle quality implementation initiative at urgent care sites on rates of appropriate antibiotic prescriptions for acute respiratory tract infections.

## Methods

This was a retrospective, observational, pre- and post- analysis conducted at four advanced urgent care (AUC) sites affiliated with a large academic medical institution from December 2023 to January 2024 (pre-implementation) and December 2024 to January 2025 (post-implementation). The AUC clinics are staffed by emergency medicine and family medicine trained attending physicians as well as advanced practice providers (nurse practitioners and physician assistants). The AUC sites conduct around 156,000 encounters per year and have capabilities in imaging and availability of medical therapies on-site. Encounters included for analysis were patients with a primary diagnosis of respiratory tract infection. Encounters for pediatric patients (<18 yr) and those immediately referred to the emergency department from urgent care were excluded.

**Figure 1.**
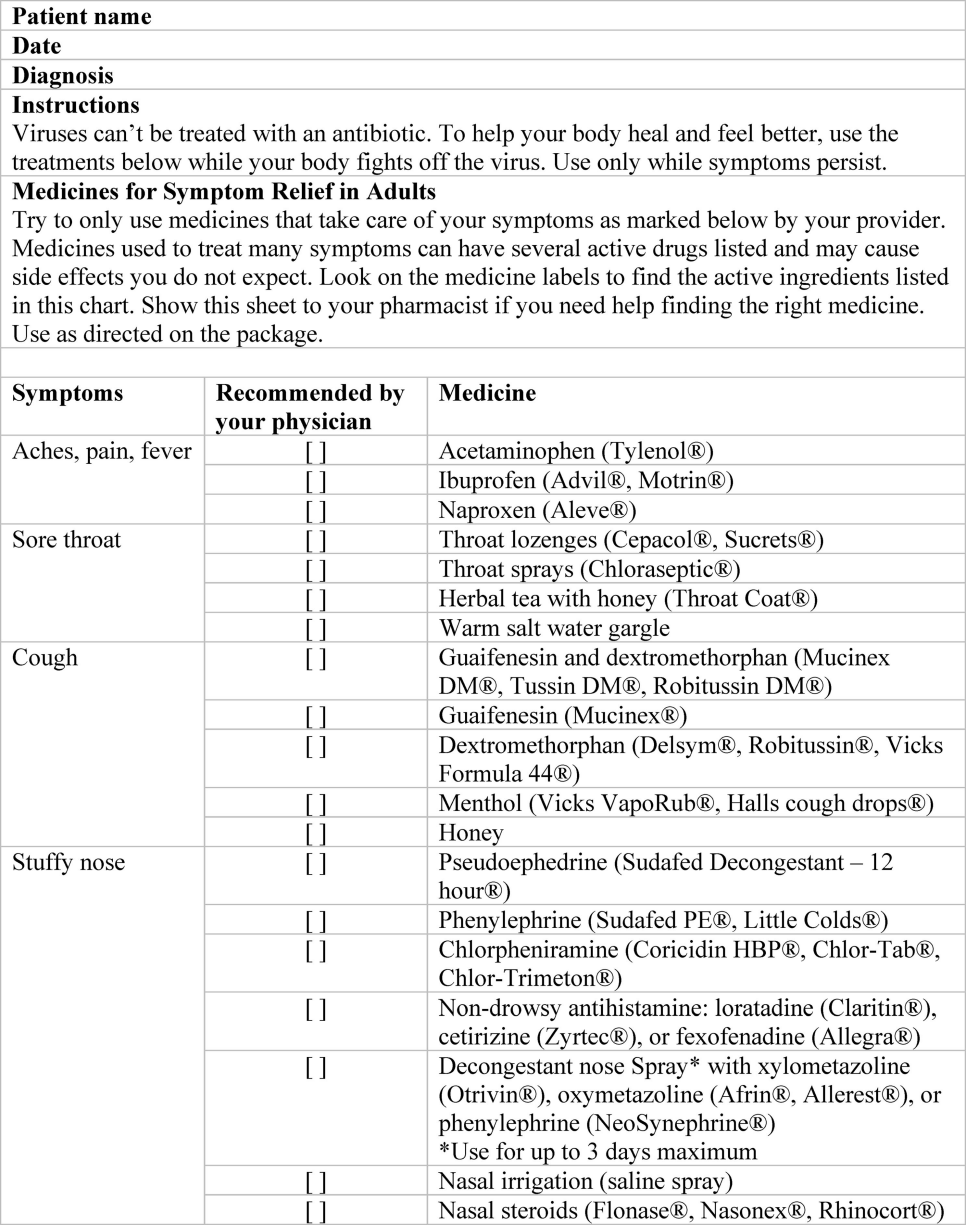
Sample over the counter prescription pad.

The antimicrobial stewardship bundle was introduced by a multidisciplinary antimicrobial stewardship team including an infectious diseases physician and an emergency medicine pharmacist team as well as physician and advanced practice provider champions from the AUC clinics. The outpatient antimicrobial stewardship team implemented similar initiatives at primary care clinics at the institution in 2021. The bundle introduced included public displays of commitment via clinician-signed stewardship posters in all AUC exam rooms, over-the-counter (OTC) medication prescription pads (available as physical pads and electronic use) for clinicians to provide to patients, and an educational campaign for clinicians (sample of OTC medication prescription pad in Figure 1). The interventions were implemented throughout October and November of 2024. Additionally, there was prior institutional guidance related to best antibiotic prescribing practices for select indications including pharyngitis, sinusitis, and otitis media aligning with national recommendations.^15^ Education focused on emphasizing the importance of antimicrobial stewardship along with reviews of resources and tools available for decision-making support. The education consisted of a lecture-style presentation created in collaboration with the antimicrobial stewardship team and presented by members of the study team. It was presented in person to the AUC attending physician group and delivered electronically via recording to the advanced practice providers.

Assessment of impact of stewardship bundle between groups (pre-implementation: December 2023 to January 2024 and post-implementation December 2024 to January 2025) was two-fold. Overall trends in antibiotic prescribing during the pre and pos-tintervention periods were assessed using a de-identified data report including all encounters at the AUC sites with an ICD-10 code for a respiratory illness and if an antibiotic was prescribed. ICD-10 codes were categorized into a three-tiered system to dictate antibiotic appropriateness for each indication: always appropriate (tier 1), sometimes appropriate (tier 2), and never appropriate (tier 3). The specific tier assigned to an indication was determined by an infectious diseases attending physician utilizing evidence-based literature.

From all visits at AUC sites during study periods, encounters in the pre- and post-implementation period were randomized and a random sample reviewed until 100 encounters of either tier 2 or tier 3 respiratory diagnosis from each pre and post-implementation period were collected. A detailed chart review from a single researcher included data abstraction from the EMR in a standardized fashion, followed by an independent check by a senior researcher on 10% of patients. Cases were assessed for appropriateness of antibiotics in accordance with guidelines. Deviations from guidelines were sent to the study ID physician for review of appropriateness. If a patient was seen multiple times during the study period, one random encounter was included.

The primary outcome was the proportion of appropriate antibiotic prescriptions for tier 2 and tier 3 respiratory tract infections (identified via ICD-10 codes). For tier 3 indications, any antibiotic prescription was considered inappropriate. For tier 2 indications, a chart review was completed to assess appropriateness by indication using institutional standards. For sinusitis, one of the following must be present: at least 10 days of symptoms, severe symptoms (fever, purulent nasal discharge, or facial pain) for at least 3 days, or patient experiencing worsening symptoms after initial improvement. For pharyngitis, this required a positive Group A Streptococcus rapid test. For otitis media, at least one severe symptom must be present: hearing loss, severe pain, and/or marked tympanic membrane erythema.^16^ For the other tier 2 indications, where explicit guidance was not provided in institutional guidelines, appropriateness of antibiotics was determined through project group consensus. If a patient presented with a tier 2 or tier 3 respiratory tract infection and was prescribed an antibiotic for a different, antibiotic-appropriate, non-respiratory indication, the antibiotic was considered appropriate. Secondary outcomes included the appropriateness of antibiotics for tier 2 diagnoses, patients with return visits for the same diagnosis within 30 days, overall antibiotic prescribing rates, and overall rate of azithromycin prescriptions.

The primary end point of overall rate of appropriate antibiotic prescriptions for tier 2 and tier 3 indications as well as secondary endpoints and nominal variables were compared using chi-squared or Fisher’s exact test, as appropriate. The students t-test or Wilcoxon rank sum test was used for continuous variables, based on normality of data. All statistical tests were evaluated for significance using an alpha level of .05. We estimated that a sample size of at least 162 patients would provide the study with 80% power, at a two-sided significance level of .05, to show improvement of appropriate antibiotic prescriptions between the pre and postimplementation periods based on anticipated incidence in the preimplementation group of 60%. Statistical analyses were performed using SAS version 9.3. The study was reviewed by the Ohio State University Institution Review Board and deemed exempt.

## Results

**Figure 2.**
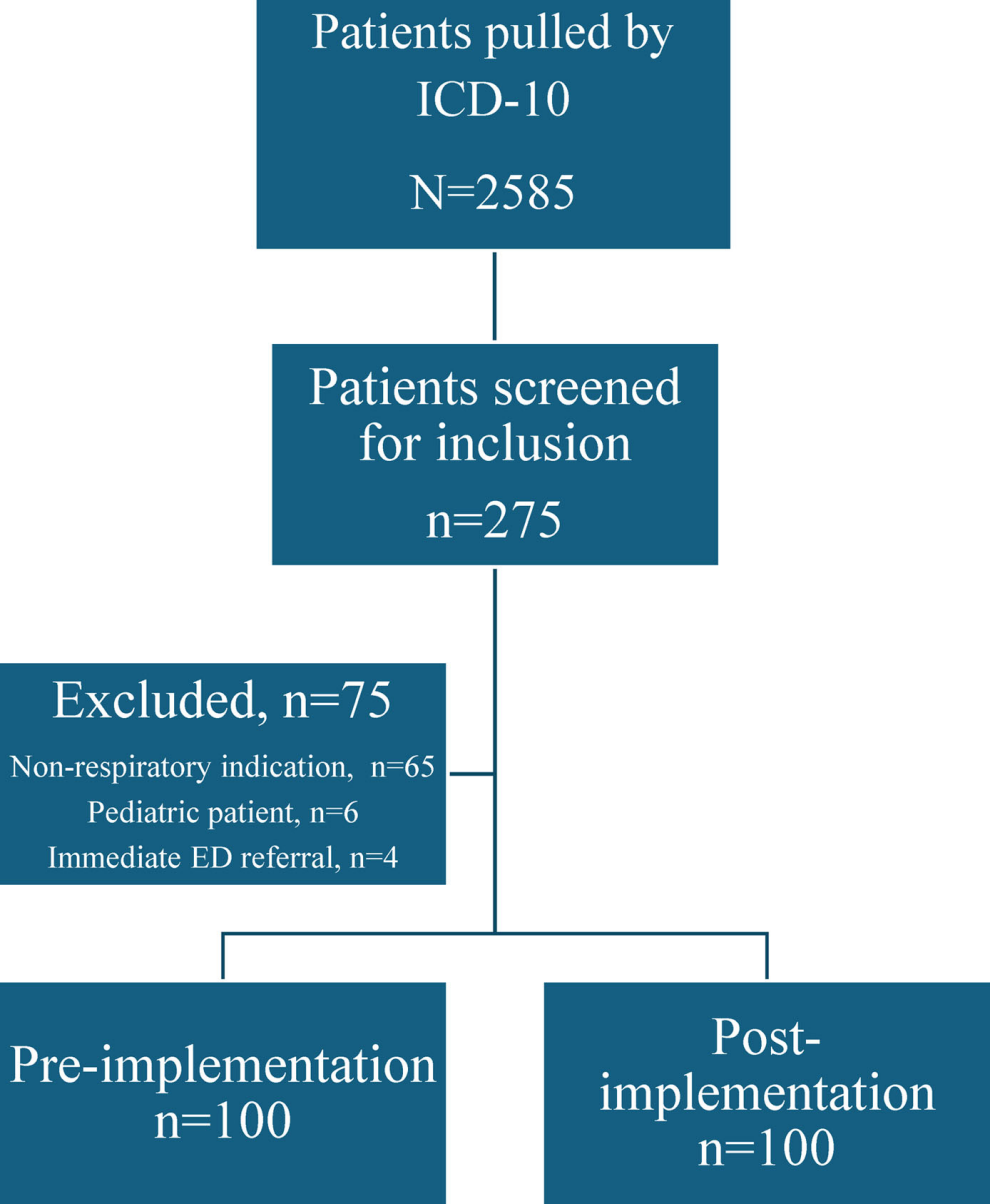
Flowchart on patients included for analysis.

A total of 2,585 patients were evaluated at AUC sites with an ICD-10 code associated with a tier 2 or tier 3 respiratory tract infection during the study periods. Of those patients, 275 were screened and 200 patients were included in the final analysis with 100 patients in each pre and postgroup (Figure 2). The most common reason for exclusion was a mismatch between ICD-10 code and documented diagnosis. Baseline characteristics were well balanced between groups with similar rates of diagnosis and days of symptoms. There was a higher proportion of patients in the postimplementation group seen by physicians and for tier 3 indications (Table 1). There was a low rate of immunosuppressed patients in both groups. Amongst patients who received a tier 3 diagnosis, eight were deemed to have an appropriate (tier 1 or tier 2) indication after chart review.

**Table 1.** Baseline demographics

Patient Characteristic	PreImplementation (n = 100)	PostImplementation (n = 100)	*p*-value
**Age, median (IQR)**	54.3 (39.0 – 68.2)	51.9 (38.8 – 67.0)	.68
**Male**	44 (44)	35 (35)	.24
**Days of symptoms, median (IQR)^a^**	7 (3 – 14)	7 (4 – 14)	.55
**Immunosuppression**	7 (7)	6 (6)	.80
**Tier 2 diagnosis**	31 (31)	19 (19)	.06
**Tier 3 diagnosis**	69 (69)	81 (81)	
**Diagnosis root name**			
** Acute Bronchitis**	15 (15)	15 (15)	-
** COPD**	6 (6)	1(1)	-
**Cough**	22 (22)	28 (28)	-
** Covid**	5 (5)	3(3)	-
** Dyspnea**	5 (5)	8(8)	-
** Influenza**	3 (3)	6 (6)	-
** Otitis Media**	2 (2)	2 (2)	-
** Pharyngitis**	16 (16)	7 (7)	-
** Sinusitis**	6 (6)	8 (8)	-
** URI**	12 (12)	11 (11)	-
** Other**	8 (8)	11 (11)	-
**Provider Type^b^**			
** Physician**	66 (66)	78 (78)	.04
** Advanced Practice Provider**	34 (34)	21 (21)	
**Previous urgent care or emergency department within 30 days**	9 (9)	12 (12)	.46

Data presented as n(%) or median (IQR).

Abbreviations: IQR, interquartile range; COPD, chronic obstructive pulmonary disease; URI, upper respiratory tract infection.

a n = 97 in the Preimplementation group and n = 98 in the Postimplementation group.

b One patient in the postimplementation group was seen by both provider types.

Following the implementation of the antimicrobial stewardship bundle to AUC sites, there was no significant difference in rates of appropriate antibiotic prescriptions for tier 2 and tier 3 respiratory infections between the pre- and post-groups (76% vs 74%, respectively; *P* = .68) (Table 2). Rates of antibiotic prescribing were 35% in the pre-implementation group and 43% in the post-implementation group (*P* = .20). There were four patients in pre-implementation group and ten patients in the post-implementation group, all with tier 3 diagnoses, who were treated with antibiotics that were ultimately deemed appropriate after retrospective chart review noting a respiratory clinical indication. One patient in the pre-implementation group and 3 patients in post-implementation group were prescribed antibiotics for non-respiratory bacterial indications, thus deemed appropriate. Among patients in the pre-implementation group, the most frequently prescribed antibiotic was amoxicillin-based products (13%) followed by doxycycline (11%). In the post-implementation group, the most frequently prescribed antibiotic was azithromycin (16%) followed by doxycycline (12%). Only five patients received prescriptions for two antibiotics.

**Table 2.** Outcomes

Outcome	Preimplementation (n = 100)	Postimplementation (n = 100)	*P*-value
Primary Outcome			
Rate of appropriate antibiotic prescriptions for tier 2 and tier 3 diagnoses	76 (76)	74 (74)	0.68
Secondary Outcomes			
Rate of appropriate antibiotic prescriptions for tier 2 indications^a^	24 (77)	18 (95)	0.13
Patients returning to AUC for a similar indication within 30 days	10 (10)	2 (2)	0.03

Data presented as n(%).

Abbreviations: AUC, advanced urgent care.

a n = 31 in the Preimplementation group and n = 19 in the Postimplementation group.

For secondary outcomes, specifically for tier 2 respiratory indications, there was a trend toward more patients with appropriately prescribed antibiotics in the post-implementation group (77% vs 95%; *P* = .13) (Table 2). Among tier 2 patients, amoxicillin-based regimens were most common in both groups. There was a higher proportion of patients returned to the AUC for a similar indication within 30 days in the pre-implementation group (10% vs 2%; *P* = .03). Only two patients were admitted to the hospital within 30 days of the initial visit for a similar indication. Overall prescribing rates analyzed from the de-identified data set increased between pre- (n = 1587) and post-implementation (n = 1981) groups (23% vs 33%; *P* = .001). This was also observed for overall azithromycin prescriptions in the same population (8% vs 14%: *P* = .001).

## Discussion

While no improvement in the rate of appropriate antibiotic prescriptions for tier 2 and tier 3 respiratory tract infections was noted, there was a numeric increase in the proportion of appropriate antibiotic prescriptions for tier 2 respiratory tract infections. We utilized a tiered system to identify antibiotic appropriateness by indication with billing codes and conducted detailed chart review to confirm antibiotic appropriateness in a sample of patients. The impact of stewardship interventions may be tier-specific, and interventions should be tailored to target more specific areas of stewardship. These findings contribute to other literature evaluating various strategies to implement antimicrobial stewardship in the urgent care setting.^8,10–14,17^

Many studies found benefits after implementing antimicrobial stewardship initiatives; however, the generalizability of a multistep intervention may not yield the same results at other institutions.^8,10–14,17^ While clinical knowledge is a component of appropriate antibiotic prescribing, there are a number of prescriber and patient factors influencing prescribing.^18,19^ The approach to improving antibiotic prescribing should be multifaceted as recommended by the CDC; however, if improvements in prescribing are not found, additional strategies or modifications should be pursued. Patel et al. evaluated the effect of a stewardship bundle on overall antibiotic prescribing rates at over 20 urgent care sites.^12^ In addition to clinician education and commitment posters, the stewardship bundle included comparative feedback for clinicians and a patient education pamphlet. Over three years (2019–2021), there was a decrease in antibiotic prescribing in adult patients seen for upper respiratory tract infections from 44% to 16%.^12^ Laude et al. conducted a study evaluating total antibiotic prescribing rates after implementing an antimicrobial stewardship bundle at urgent care centers over a longer period (November 2016—June 2019) and found continued improvement in antibiotic prescribing rates and azithromycin prescribing rates with a reduction from 54.7% to 35% throughout their study period.^14^ Unlike our study, there was a component of ongoing feedback via a real-time antibiotic utilization dashboard and targeted education based on the dashboard. The longer study period as well as the education strategy used in their study allowed for more education and adoption of resources, which may have increased utilization rates by providers. These findings emphasize the complexity of antimicrobial stewardship efforts and highlight the need for adaptable strategies accounting for the multifactorial influences on prescribing behavior.

In our study, two periods of time were selected to coincide with higher rates of respiratory viral infections in the community thus targeting times where inappropriate antibiotic prescribing could be most impacted. However, there were notable differences between the periods with the post-implementation period noting significantly higher rates and severity of influenza and concerns for mycoplasma infections in the local community in the post-implementation period.^20,21^ Viral trends may have variable effects on antibiotic prescribing as noted by Bizune et al. who observed a decrease in antibiotic prescriptions during the COVID-19 pandemic but an increased rate of azithromycin prescriptions.^22^ In chart review there was trend toward patient concerns for exposure to *Mycoplasma* and suggested concerns of a local community outbreak. If a patient did have potential exposure to *M. pneumoniae* with upper respiratory tract symptoms, antibiotic therapy is still not indicated without radiographic evidence of pneumonia. Rates of antibiotic prescriptions for laboratory confirmed or presumed *M*. *pneumonia* were not explicitly collected in this study; however, an increase in both azithromycin and doxycycline prescriptions was noted in the post-period. These findings are exploratory and warrant further investigation. Therefore, the increased rate of overall antibiotic prescriptions and azithromycin prescriptions in our study may have been confounded by local epidemiological patterns during the post-implementation period.

Limitations of this study include its retrospective design at a single institution perhaps limiting generalizability to other healthcare settings. We aimed to overcome this through broad inclusion criteria. This was the first organized outpatient stewardship initiative to be done at the AUC clinics despite initiatives being in place at other sites within the system. Due to the nature of the electronic health record, we were unable to assess revisits at urgent care sites outside of the institution’s network. Patient education and expectations were addressed via the commitment posters and OTC medication prescription pad; however, these may be considered passive educational tools. Active education as a component of the patient visit was not tracked and should be considered in the future.^23,24^ Our study did not collect data regarding utilization rate of resources which would be key for future initiatives to understand barriers to utilization of evidence-based interventions. Lastly, this study did not evaluate the appropriateness of antibiotic prescriptions based on agent selection, dose, or duration and only assessed for an appropriate indication.

Our study found no improvement in appropriate antibiotic prescriptions after implementation of a stewardship bundle, highlighting the need for ongoing efforts to identify the most effective method for outpatient stewardship. Future initiatives should focus on targeting real-time infectious trends and monitoring trends over longer periods of time for full adoption of resources. Additionally, provider variability should be explored to consider more targeted interventions in such a large pool of providers. Potential solutions include just-in-time educational initiatives for emerging infections and a prescribing dashboard. The prescribing dashboard could be organized by inappropriate antibiotic prescribing based on tiered diagnoses to allow for targeted improvement plans. While not all detailed decision making would be included using billing codes alone to categorize by tiers, in the sample reviewed for this project, only eight patients with a tier 3 diagnosis were ultimately found to have an appropriate (tier 1 or tier 2) indication after chart review. Strategies to minimize diagnosis shifting should be considered for more long-term monitoring initiatives.

While this study did not demonstrate a significant improvement in appropriate antibiotic prescribing after implementation of an antimicrobial stewardship bundle at AUC clinics, it highlights the challenges in implementing antimicrobial stewardship initiatives in the outpatient setting. Factors such as analyzing a snapshot of time rather than a prolonged period and limited adoption period may have contributed to these findings. Future initiatives should focus on tailored interventions acknowledging tier-specific interventions, emerging infectious trends and providing continuous feedback and education to further optimize antibiotic prescribing in urgent care settings.

## Data Availability

Due to institutional restrictions, data is not publicly available.
